# Team functioning and implementation of innovations in healthcare and human service settings: a systematic review protocol

**DOI:** 10.1186/s13643-021-01747-w

**Published:** 2021-06-26

**Authors:** Elizabeth A. McGuier, David J. Kolko, Mary Lou Klem, Jamie Feldman, Grace Kinkler, Matthew A. Diabes, Laurie R. Weingart, Courtney Benjamin Wolk

**Affiliations:** 1 University of Pittsburgh School of Medicine, 3811 O’Hara Street, Pittsburgh, PA 15213 USA; 2 University of Pittsburgh, Health Sciences Library System, 3550 Terrace Street, Pittsburgh, PA 15261 USA; 3 University of Pittsburgh Medical Center, 3811 O’Hara Street, Pittsburgh, PA 15213 USA; 4 University of Pennsylvania, 3535 Market Street, 3rd floor, Philadelphia, PA 19104 USA; 5 Tepper School of Business, Carnegie Mellon University, 5000 Forbes Ave, Pittsburgh, PA 15213 USA

**Keywords:** Team; Teamwork, Implementation, Systematic review, Healthcare

## Abstract

**Background:**

Healthcare and human services increasingly rely on teams of individuals to deliver services. Implementation of evidence-based practices and other innovations in these settings requires teams to work together to change processes and behaviors. Accordingly, team functioning may be a key determinant of implementation outcomes. This systematic review will identify and summarize empirical research examining associations between team functioning and implementation outcomes in healthcare and human service settings.

**Methods:**

We will conduct a comprehensive search of bibliographic databases (e.g., MEDLINE, PsycINFO, CINAHL, ERIC) for articles published from January 2000 or later. We will include peer-reviewed empirical articles and conference abstracts using quantitative, qualitative, or mixed methods. We will include experimental or observational studies that report on the implementation of an innovation in a healthcare or human service setting and examine associations between team functioning and implementation outcomes. Implementation outcomes of interest are acceptability, adoption, appropriateness, cost, feasibility, fidelity, penetration, and sustainability. Two reviewers will independently screen all titles/abstracts, review full-text articles, and extract data from included articles. We will use the Mixed Methods Appraisal Tool to assess methodological quality/bias and conduct a narrative synthesis without meta-analysis.

**Discussion:**

Understanding how team functioning influences implementation outcomes will contribute to our understanding of team-level barriers and facilitators of change. The results of this systematic review will inform efforts to implement evidence-based practices in team-based service settings.

**Systematic review registration:**

PROSPERO CRD42020220168

**Supplementary Information:**

The online version contains supplementary material available at 10.1186/s13643-021-01747-w.

## Background

Team-based approaches to care, in which multiple providers work collaboratively with patients and families toward shared goals, are increasingly common across a range of healthcare and human service settings [1–5]. Occurring alongside the shift to team-based care is an increasing emphasis on evidence-based practice in healthcare and human services. In team-based service settings, implementing evidence-based practices and other innovations requires teams to change processes and behaviors to respond to new demands, yet little research has examined how team functioning influences implementation [6, 7].

Team functioning can be defined as how teams think, feel, and act [8–12]. Team functioning refers to processes and emergent states that may be affective, behavioral, or cognitive [8, 9]. Cognitive and affective states emerge from behaviors and interactions between team members and have reciprocal effects on these behaviors [8, 9]. Affective aspects of team functioning include cohesion, trust, respect, and collective efficacy. Behavioral processes include communication, coordination, conflict resolution, information sharing, and decision-making. Lastly, cognitive aspects include knowledge, shared mental models, and diversity in members’ expertise [9, 12–19]. Better team functioning is associated with better team performance in diverse work settings [8, 20, 21].

Research on teams in healthcare has focused primarily on the influence of team functioning on service outcomes (i.e., patient safety, efficiency, effectiveness, equity, patient-centeredness, and timeliness [22]). Better team functioning is associated with greater patient safety and better patient outcomes [23, 24], and there is increasing evidence that interventions to improve the functioning of healthcare teams improve patient safety and outcomes [24–28]. Less attention, however, has been paid to the role of teams in the implementation of evidence-based healthcare interventions.

**Fig. 1:**
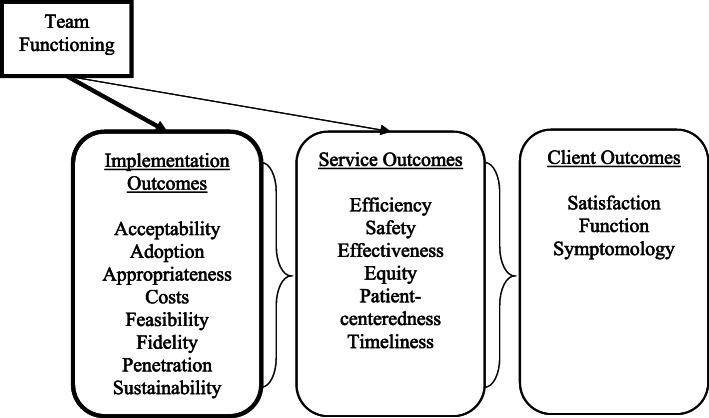
Conceptual model (adapted from Proctor et al. [31]). Note: Dark outlines indicate the focus of this systematic review

Implementation of evidence-based practices is a multi-phased process that unfolds over time and is influenced by determinants (i.e., barriers and facilitators) at multiple levels. This systematic review is guided by the Exploration, Preparation, Implementation, and Sustainment (EPIS) determinant framework [29, 30] and Proctor and colleagues’ implementation outcomes framework [31]. EPIS specifies four phases—Exploration, Preparation, Implementation, and Sustainment—and describes inner context (i.e., within the organization) and outer context (i.e., external to the organization) determinants and mechanisms, as well as innovation qualities and bridging factors (linking outer and inner contexts) that affect implementation [29, 30]. Implementation outcomes, as defined by Proctor et al. [31], include acceptability, adoption, appropriateness, costs, feasibility, fidelity, penetration, and sustainability of the evidence-based practice being implemented [31]. They are distinct from and precede traditional effectiveness or patient-level outcomes (Fig. 1).

There is extensive evidence that characteristics of individual providers and organizations influence the implementation process [7, 30, 32–35]. However, team-level determinants of implementation have received less research attention. Team functioning has been shown to impact service and patient outcomes in healthcare [23, 24] and may impact these outcomes in part through its impact on implementation outcomes. Because teams must change processes and behaviors to implement a new practice, affective, behavioral, and cognitive aspects of team functioning are likely to influence the implementation process and affect implementation outcomes because of the need for teams to change. Figure 1 illustrates how team functioning may impact outcomes in healthcare and human service settings. The goal of this systematic review is to identify and summarize empirical research examining associations between team functioning and implementation outcomes when evidence-based practices and other innovations are implemented in healthcare and human service settings.

## Methods

The protocol for this systematic review has been registered in the international prospective register of systematic reviews (PROSPERO; registration number: CRD42020220168) and is reported in accordance with the guidance provided in the Preferred Reporting Items for Systematic Review and Meta-Analysis Protocols (PRISMA-P) statement ([36, 37]; see checklist in Additional File 1).

### Eligibility criteria

Studies will be selected based on study design, setting, study characteristics, and outcomes, as described below.

#### Study design

We will include peer-reviewed empirical articles and conference abstracts using quantitative, qualitative, or mixed methods. We will include both observational (e.g., cross-sectional studies, cohort studies) and experimental studies (e.g., randomized controlled trials, nonrandomized trials). Study protocols, reviews (including meta-analyses), and commentaries will be excluded.

#### Setting

We will include studies conducted in healthcare or human service settings. Examples include primary care practices, hospitals, specialty clinics, treatment centers, long-term care facilities, group homes, correctional facilities, child welfare or youth services, aging services, and schools. We will exclude studies conducted in higher education settings unless specific to healthcare (e.g., a study conducted in a university health clinic).

#### Study characteristics

We will include studies that describe and report on the implementation of an innovation or intervention to improve patient care (e.g., guideline, evidence-based treatment). We will exclude studies of interventions designed to improve teamwork (e.g., team building or team training interventions) and studies of teams created to implement an innovation (e.g., implementation teams, quality improvement teams). Eligible studies must assess and report on at least one aspect of team functioning and test its association with at least one implementation outcome. For this review, we define team functioning as how teams think, feel, and act and include studies that measure affective, behavioral, or cognitive processes or emergent states. Implementation outcomes are defined below.

#### Outcomes

We will include studies that assess and report at least one implementation outcome, as defined by Proctor and colleagues [31]. Primary outcomes of interest are acceptability (i.e., perceptions that innovation is acceptable), adoption (i.e., decision to use innovation), appropriateness (i.e., perceived fit of innovation), costs of implementation effort, feasibility of the innovation, fidelity (i.e., degree to which innovation is implemented as designed), penetration/reach (i.e., extent to which innovation is integrated in setting; proportion of eligible recipients who receive innovation), and sustainability (i.e., extent to which innovation is maintained over time). We will include studies using common synonyms or other terms to describe implementation outcomes (e.g., extent of implementation, implementation quality). Results for each implementation outcome will be reported separately.

### Information sources and search strategy

An experienced librarian (MLK) will develop bibliographic database search strings using controlled vocabulary, e.g., Medical Subject Headings (MeSH), and natural language terms to represent the concepts of “team functioning” and “implementation of evidence-based practice.” We will search the following electronic databases: Ovid MEDLINE, Ovid PsycINFO, EBSCOhost CINAHL, and EBSCOhost ERIC. A draft search strategy for Ovid MEDLINE is provided in Additional File 2. A publication date limit of 2000 to current will be applied to all searches. In 2000, the Institute of Medicine published the seminal report *To Err is Human*, which identified teamwork as important to reducing medical errors and initiated a period of increasing attention to teamwork in healthcare [23]. The past 20 years also includes the rapid development of implementation science as an independent field of research, marked by the founding of *Implementation Science* in 2006 and the first Annual Conference on the Science of Dissemination and Implementation in Health in 2008. Articles published in languages other than English will be translated using Google Translate [38].

After completing the above database searches, we will also search available conference abstracts from two leading implementation conferences (Annual Conference on the Science of Dissemination & Implementation in Health 2008–2020; Society for Implementation Research Collaboration 2011–2019). Lastly, we will hand-search the reference lists of included articles, perform a cited reference search for included articles in the Web of Science and Scopus databases, and consult content experts to identify additional relevant articles. Searches will be re-run prior to the final analysis, with any additional identified studies retrieved and considered for inclusion.

### Selection process

DistillerSR software will be used to store search results and conduct reference screening. After removing duplicates, two independent reviewers will screen titles and abstracts for inclusion. We will pilot screening forms with at least 10 articles and refine forms before screening. Discrepancies in inclusion/exclusion determinations will be resolved through re-review, discussion between reviewers, and if needed, review of the title/abstract by a third reviewer. Next, two independent reviewers will review full texts to determine if the article should be included or excluded. Again, we will pilot forms with at least 10 articles and refine forms before beginning the review phase. Disagreements will be resolved through discussion between reviewers, and if needed, consultation with a third author. We will assess inter-rater reliability (e.g., Cohen’s kappa, percent agreement) for the title/abstract screening and full-text review phases. We will document reasons for exclusion and use a PRISMA flow diagram to present the number of identified, included, and excluded articles.

### Data extraction

We will use DistillerSR software to extract relevant data, including basic study information (e.g., authors, publication year, funding source), study aim(s), study setting, sample characteristics (e.g., eligibility criteria, sample size, participant demographics), study type and design, innovation implemented, implementation methods, assessment timepoints, measures of team functioning, implementation outcome(s), statistical analysis methods, and results. We will pilot the data extraction form with at least 10 articles and refine the form before beginning data extraction. Data extraction will be performed by one reviewer and independently verified by a second. Discrepancies will be resolved through re-review of the original study, discussion, and if needed, consultation with a third author. Authors will be contacted for unreported data.

### Quality and risk of bias assessment

After data has been extracted from all relevant studies, we will assess the methodological quality of each included study using the Mixed Methods Appraisal Tool (MMAT) [39]. This tool provides criteria to evaluate the quality of qualitative, quantitative, and mixed methods studies. Multiple publications on the same study will be assessed as a group. Each study will be assessed by one reviewer and independently verified by a second. Discrepancies between reviewers will be resolved through discussion and consultation with a third author when needed to reach consensus. Quality appraisal results will be summarized in a table and described in the narrative synthesis of findings.

### Data synthesis

We plan to conduct a narrative synthesis of included articles following guidelines for the reporting of synthesis without meta-analysis (SWiM [40]). If more than five studies test associations between a specific aspect of team functioning and the same implementation outcome, we will conduct meta-analyses using random-effects models. Data extracted from included studies will be presented in tables organized by dimensions of team functioning (i.e., affective, behavioral, or cognitive). Tables will include characteristics of the setting (e.g., healthcare vs. human services) and team (e.g., size, stability) to allow for consideration of heterogeneity in findings. Tables will also include standardized metrics of association (e.g., correlation coefficients) for quantitative studies. Review findings will be presented in succinct tables that include lists of individual studies contributing to the finding, the range and distribution of quantitative results, summaries of qualitative findings, and ratings of evidence quality (described below).

### Confidence in cumulative evidence

We will assess the strength of the overall body of evidence using GRADE [41] and GRADE-CERQual [42]. Both approaches result in ratings of high, moderate, low, or very low quality of evidence. GRADE ratings are based on (1) risk of bias in individual studies, (2) imprecision, (3) inconsistency across studies, (4) indirectness, and (5) publication bias. Similarly, GRADE-CERQual is designed for qualitative studies, and ratings are based on (1) methodological limitations of individual studies, (2) coherence, (3) adequacy (i.e., richness and quantity) of data, and (4) relevance to review question. Two independent reviewers will make ratings, and discrepancies between reviewers will be resolved through discussion and consultation with a third author when needed to reach consensus.

## Discussion

Despite the increasing use of team-based care in healthcare and human services, little research has examined team-level determinants of implementation of evidence-based practices. Affective, behavioral, and cognitive aspects of team functioning are likely to affect the ways in which teams respond to change efforts and therefore impact implementation outcomes. We anticipate that better team functioning (e.g., high cohesion, effective communication) will be associated with better implementation outcomes, while problems in team functioning (e.g., poor conflict resolution, low trust) will negatively impact implementation outcomes.

This systematic review will provide a comprehensive summary of empirical research on associations between team functioning and implementation outcomes in healthcare and human service settings. Results will be reported in accordance with the PRISMA 2020 statement [43] and disseminated through conference presentations and publication in a peer-reviewed journal. Any changes to the protocol described here will be added in an amendment in PROSPERO and described in the final manuscript. Limitations of this review include the possibility of unintentionally omitting some relevant studies (e.g., studies that refer to work units or groups instead of teams). To reduce this risk, we will consult with content experts, search abstracts from prominent implementation conferences, and search reference lists of included articles. Although inclusion of qualitative and mixed methods studies is likely to increase the time required to identify relevant articles [44], they will be included because of their widespread use in implementation research [45–47]. Another likely challenge will be the operationalization and measurement of team functioning and implementation outcomes. There is likely to be significant variability in the specific domains of team functioning assessed by individual studies. Similarly, there is variability in implementation outcomes reported across studies and considerable unevenness in the availability and psychometric quality of implementation outcome measures [48]. Variations in how team functioning is defined and measured and variations in implementation outcomes may contribute to inconsistency across studies and hinder our ability to draw conclusions.

The findings of this systematic review will highlight gaps in our understanding of team-level influences on implementation to consider in future research. Findings may also inform the development and selection of implementation strategies to target team-level mechanisms. Understanding how team functioning influences implementation outcomes will contribute to our understanding of mechanisms of change and inform the use of targeted implementation strategies in team-based service settings.

## Supplementary Information

**Additional file 1.** PRISMA-P 2015 Checklist.

**Additional file 2.** MEDLINE Search Example.

## Data Availability

Not applicable.

## Abbreviations

EPIS: Exploration, Preparation, Implementation, and Sustainment
GRADE: Grading of Recommendations Assessment, Development, and Evaluation
GRADE-CERQual: Grading of Recommendations Assessment, Development, and Evaluation-Confidence in the Evidence from Reviews of Qualitative research
MeSH: Medical Subject Headings
MMAT: Mixed Methods Appraisal Tool
PRISMA: Preferred Reporting Items for Systematic Reviews and Meta-Analyses
PRISMA-P: Preferred Reporting Items for Systematic Review and Meta-Analysis Protocols
