# Supplementary material for: Team functioning and implementation of innovations in healthcare and human service settings: a systematic review protocol
Source: Syst Rev. 2021 Jun 26;10:189. doi: 10.1186/s13643-021-01747-w (PMC8236140; doi:10.1186/s13643-021-01747-w)
Supplement: Supplementary file 2 — Additional file 2. MEDLINE Search Example. [file 13643_2021_1747_MOESM2_ESM.docx]

**Additional File 2**

*MEDLINE Search Example*

| Search Terms |
| --- |
| ((team adj1 (learning or performance or collaboration or building or leader*)) or (team adj2 (functioning or effectiveness or process* or stability or mental model* or climate or coherence or knowledge or cohesion or cohesiveness or affect or mood or emotion* or dynamic* or efficacy or conflict* or norms or coordination or cooperation or communication or competenc* or regulation)) or ((team work or teamwork) adj2 (functioning or effectiveness or process* or stability or mental model* or climate or coherence or knowledge or cohesion or cohesiveness or affect or mood or emotion* or dynamic* or efficacy or conflict* or norms or coordination or cooperation or communication or competenc* or regulation))).ti,ab. or (*Patient Care Team/ and (functioning or effectiveness or processes or stability or mental model* or climate or coherence or learning or knowledge or cohesion or cohesiveness or affect or mood or emotion* or dynamic* or efficacy or conflict* or norms or coordination or cooperation or communication or competenc* or regulation).ti,ab.)  AND  (implementation science/ or "Diffusion of Innovation"/ or implementation science.jn. or implementation.mp. or innovation*.mp. or exp Evidence-Based Practice/ or evidence based.mp. or Guideline Adherence/ or Guidelines as Topic/ or Practice Guidelines as Topic/ or (best practice* or guideline* or dissemination or knowledge translation).ti,ab. or (Electronic Health Records/ or electronic health record*.ti,ab. or electronic medical record*.ti,ab. or Cognitive Behavioral Therapy/ or *Checklist/)) |
